# Effects of UV-B light exposure during automatic milking on vitamin D levels in Holstein Friesian cows

**DOI:** 10.3389/fvets.2024.1433230

**Published:** 2025-01-15

**Authors:** Jaka Jakob Hodnik, Marko Jankovec, Jožica Ježek, Žiga Krušič, Stefan Mitterhofer, Jože Starič

**Affiliations:** ^1^Clinic for Reproduction and Large Animals-Section for Ruminants, Veterinary Faculty, University of Ljubljana, Ljubljana, Slovenia; ^2^Scottish Centre for Production Animal Health and Food Safety, University of Glasgow, Glasgow, United Kingdom; ^3^Laboratory of Photovoltaics and Optoelectronics, Faculty of Electrical Engineering, University of Ljubljana, Ljubljana, Slovenia; ^4^Consultant, Bonn, Germany

**Keywords:** ultraviolet-B light, cattle, 25-hydroxyvitamin D, milk yield, skin, hair, color, blood

## Abstract

Vitamin D is essential for cattle and can be synthesized in the skin under ultraviolet irradiation. This study investigated the effects of narrow-band UV-B irradiation during automatic milking on blood vitamin D concentration and the influence of hair and black skin areas on cutaneous vitamin D synthesis in Holstein Friesian cows. Fifty-one cows were stratified by milk yield, days after calving, and percentage of black skin, then divided into three groups: shaved and irradiated (80 J/m^2^), unshaved and irradiated (129–305 J/m^2^), and a control group. A custom UV-B light (peak radiation at 295 nm) was installed in the milking robot. Blood 25-hydroxyvitamin D (25(OH)D) levels were measured at baseline, and 7, 30, and 60 days post-exposure using an enzyme-linked fluorescent assay. UV-B exposure significantly (*p* < 0.001) increased 25(OH)D levels in shaved (13.4 ng/mL), unshaved (10 ng/mL), and control groups (5.1 ng/mL). Despite receiving less than half the UV-B dose, the shaved group had superior 25(OH)D synthesis compared to the unshaved group (*p* < 0.05), highlighting hair’s role in reducing UV-B absorption. Cutaneous synthesis correlated with black skin area in shaved cows but not in unshaved cows. UV-B irradiation also increased daily milk production by 2.2 kg (shaved) and 2.9 kg (unshaved) compared to controls (*p* < 0.001). UV-B exposure during automatic milking offers a novel, non-disruptive method for enhancing vitamin D levels in dairy cows.

## 1 Introduction

Vitamin D is an essential vitamin for calcium and phosphorus metabolism, bone homeostasis, and immunity (1, 2). Cattle can obtain vitamin D from feed or through cutaneous synthesis under the influence of UV-B irradiation (3). Vitamin D must be hydroxylated twice, once in the liver to form 25-hydroxyvitamin D (25(OH)D) and the second time in the kidneys to form 1,25-dihydroxyvitamin D (1,25(OH)_2_D) (4). Dietary vitamin D comes in two forms: vitamin D_2_ and D_3_, which are of fungal and animal/synthetic origin, respectively, however cattle cannot utilize dietary vitamin D_2_ as effectively as vitamin D_3_ (5, 6). Cattle synthetize vitamin D_3_ across their entire skin surface in a dose dependent manner (3, 7). In the summer, cattle on pasture can produce enough vitamin D_3_ to meet their physiological needs (7); however, most dairy cows are kept indoors for at least part of their production cycle. In humans, cutaneous pre-vitamin D production is optimal at UV wavelengths between 295 and 300 nm (8). Cattle experience similar seasonal variations of vitamin D levels to humans which suggests their production is optimal at the same wavelengths (9, 10). In the winter months these wavelengths are filtered out by the thicker layer of atmospheric ozone at mid-latitude locations due to a change in the sun’s position; therefore, autogenous vitamin D synthesis is insufficient (11).

Previous studies have demonstrated that cattle can increase their blood 25(OH)D levels when irradiated with an artificial UV source. However, these studies were carried out in an experimental setting that is impractical for use on commercial farms (12, 13). To the authors’ knowledge no study has attempted to use an artificial, narrow-band UV-B source for enhanced autogenous vitamin D production in a commercial setting.

In calves, a darker hair coat has been associated with lower 25(OH)D concentrations (14) however no study has demonstrated this phenomenon in adult cattle (7, 12, 13). Hair provides protection from harmful UV irradiation but also affects vitamin D production. It has been shown that shorn sheep have higher 25(OH)D levels compared to their unshorn counterparts (15). Our previous study showed that the dose of UV-B reaching the skin of cattle was linearly dependent on hair length (16). No study has investigated whether this also affects cutaneous vitamin D_3_ production in cattle.

The main objective of this study was to investigate the effects of artificial narrow band UV-B irradiation during automatic milking on cutaneous vitamin D_3_ production in a commercial dairy setting. In addition, we investigated the effects of black area of skin and hair, and presence of hair on vitamin D_3_ production.

## 2 Materials and methods

### 2.1 Animals

The study was approved by the Commission for Animal Welfare at the Veterinary Faculty, University of Ljubljana, on March 25, 2020. The study was conducted on a single commercial dairy farm that comprised 68 Holstein Friesian cows housed year-round in an indoor freestall system. Milking was carried out using a free flow automatic milking system Merlin 225 (Fullwood Packo, Ellesmere, United Kingdom) and the average milk yield over a single lactation cycle was 8,314 kg. Cows were dried off 60 days prior to the estimated calving date. Cows were included in the study if they were lactating and were not scheduled to be dried-off in the next 2 months. Fifty-one cows met the inclusion criteria. Each cow was photographed from above and the images were analyzed using the software ImageJ (National Institutes of Health, United States) to measure the percentage of black area on the back between the withers and hook bones. The percentage of black area of the hair coat was assumed to be the same as the skin. Cows were then randomly divided into three groups of 17 using stratified random sampling based on 305-day milk yield in the previous lactation or the average 305-day milk yield of their dams for primiparous cows (above or below the herd average), the percentage of black area on their back (above or below 50%), and days in milk (above or below 30 days). During the study four cows were culled from the herd, one from the shaved group and three from the unshaved group. Another cow was excluded from the analysis because it developed abomasal displacement prior to the start of the study. The study was conducted for 60 days from March to May 2022.

### 2.2 Feed

The cows were fed a partial mixed ration (PMR) twice daily (Table 1). In addition to the PMR, each cow received 0.5 kg of a concentrate feed K-22 (Jata Emona d. o. o., Ljubljana, Slovenia) during automatic milking. High-yielding cows received an additional 1 kg of K-TOP (Jata Emona d. o. o., Ljubljana, Slovenia) and 1 kg of K-TOP-50 (Jata Emona d. o. o., Ljubljana, Slovenia) in a separate automatic feeder for every 10 kg of milk they produced above a daily benchmark of 20 kg. The compositions of the concentrate feeds are shown in Table 2. The number of times that extra concentrates for high milk yield were given was similar between groups (357 shaved group, 388 unshaved group and 346 control group). The PMR was sampled and analyzed on three occasions: before the start, 1 and 2 months after the start of the study in accordance with Commission regulation 152/2009. Feed samples were taken from the feed bunk immediately after the PMR was delivered. In addition to the proximate analysis performed at the Institute of Food Safety, Feed and Environment, Veterinary faculty, University of Ljubljana (Ljubljana, Slovenia) samples were sent to Mérieux NutriSciences Chelab (Resana, Italy), where vitamin D_3_ concentrations were measured using high-performance liquid chromatography-mass spectrometry (HPLC-MS; Table 3).

**Table 1:** Composition of partial mixed ration fed daily per cow in two meals.

Component	Fresh weight (kg)
Wheat straw	0.5
Corn silage	20
Grass silage	26
Corn	0.8
Soybean meal	0.3
Canola crops	0.3
Fat powder	0.1
Beer dregs	1
K-mix-PASS*	0.8
Rumisal-10*	0.15
Limestone	0.08
Bicarbonate	0.150
Complett EXTRA-Keragen®+	0.1
Total weight	50.280

*Manufacturer: Jata Emona d.o.o., Ljubljana, Slovenia; +Manufacturer Josera d.o.o., Ljubljana, Slovenia.

**Table 2:** Nutritional composition of concentrate feeds.

Component	K-22*	K-TOP*	K-TOP-50*
Crude protein (% DM)	22	10	21
Crude fat (% DM)	2.2	7.2	5.4
Crude fiber (% DM)	7.8	5.1	6.4
Crude ash (% DM)	6.6	6.5	7.9
Calcium (% DM)	0.85	1.2	1.2
Phosphorus (% DM)	0.65	0.4	0.7
Sodium (% DM)	0.25	0.5	0.3
Magnesium (% DM)	0.3	0.4	0.5
Vitamin D_3_ (IU/kg)	1,000	0	1,000

*Manufacturer: Jata Emona d. o. o., Slovenia.

**Table 3:** Results of the proximate nutritional analyses and vitamin D_3_ concentrations in the partial mixed ration.

	Day 0	Day 30	Day 60
Moisture (%)	63.2	66.9	64.5
Crude protein (% DM)	14.7	13.7	9.7
Crude fat (% DM)	8.3	3.7	2.6
Crude fiber (% DM)	20.8	24.7	19.4
Ash (% DM)	8	7.9	5.5
Vitamin D3 (IU/kg)	261 ± 72	294 ± 81	227 ± 62
Vitamin D3 (IU/day)	13,097	14,753	11,391

### 2.3 UV light

**Figure 1:**
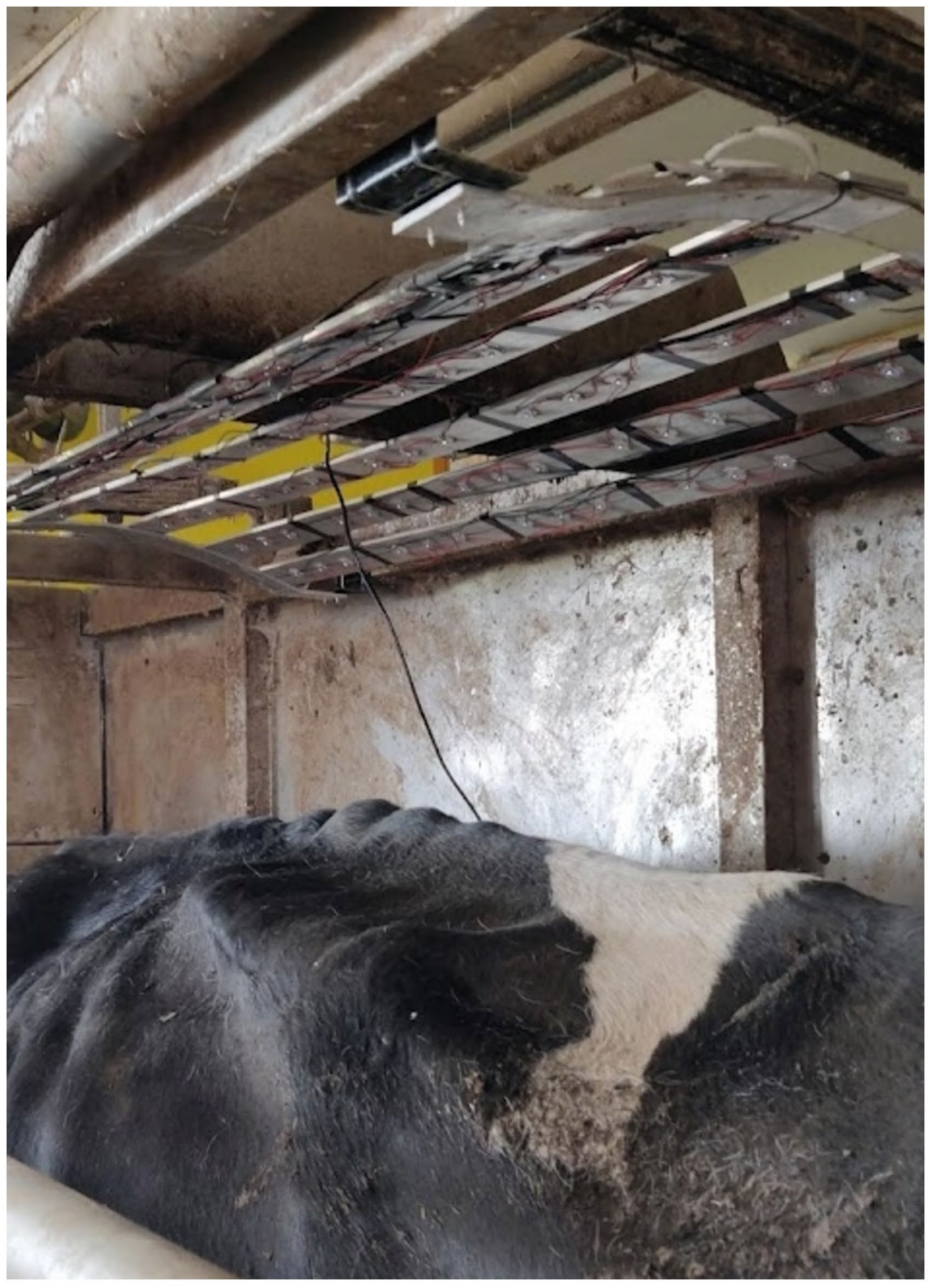
Irradiation of a cow with the narrowband UV-B LED light incorporated into the Merlin 225 automatic milking system (Fullwood Packo, Ellesmere, United Kingdom).

A custom-made UV light consisting of 90 LEDs Houkem-SMD 3535–290-300 nm (Dongguan Houke Electronic Co., Ltd., Guangdong, China) mounted in six rows on a curved aluminum frame (40 cm X 120 cm) was used in this study. The spectral power of the UV light was determined using a spectrophotometer HR2000+ (Ocean Optics Inc., Dunedin, FL, United States). The total output power of the light was 295.2 mW with a peak power at 295 nm, at 40 to 50 cm from the cows’ back. The light was installed into the Merlin 225 automatic milking system (Fullwood Packo, Ellesmere, United Kingdom) in such a way that it irradiated only the backs of cows as they were being milked (Figure 1). Cows were allowed to enter the robot every 8 h. The milking durations and number of visits to the robot per cow were not recorded however on average each animal was milked for 17.55 min per day.

### 2.4 Treatment

Each of the three groups of cows were randomly assigned to an irradiation protocol. The irradiation doses were selected based on the minimal erythema doses of cattle determined in our previous study (16) and Holick’s rule for safe daily UV exposure for vitamin D production, which states that humans need to expose ¼ of their skin surface to ¼ of their minimal erythema dose to cover their daily requirements (17). The daily irradiation process was automated: cows were identified by their leg-mounted transponders using Fullwood MerlinView software (Fullwood Packo, Ellesmere, United Kingdom) and LabVIEW: Advanced Programming Technique program (National Instruments, Austin, Texas, United States) was used to administer the daily dose of UV-B light. The automatic milking system also recorded the daily milk yield of the cows. The first group had their backs shaved 30 cm from the midline between the withers and the hook bones and were irradiated with a daily dose of 80 J/m^2^. The second group was irradiated with a maximal dose that daily automatic milking time allowed but did not exceed 360 J/m^2^. The third group served as a control group.

### 2.5 Sampling

Blood samples were taken from the cows at four timepoints: day 0 (prior to initial irradiation) and 7, 30 and 60 days after the start of irradiation. Blood samples were collected between 9 and 10 AM from the tail vein (*vena caudalis mediana*) into plain and EDTA tubes (BD Vacutainer®, Franklin Lakes, New Jersey, United States). Samples were transported to the laboratory within 1–2 h and hematology was performed the same day. The blood in plain tubes was allowed to clot overnight at room temperature (22°C). Samples were then centrifuged twice for 10 min at 1500 x g then the serum was removed, divided into three aliquots and frozen at −20°C until analysis. At the first sampling timepoint (day 0), hair samples were plucked from the dorsal surface of the lumbar transverse processes. Three randomly selected hairs were measured to determine the average hair length. The procedure was repeated in the unshaved group at the last sampling timepoint (60 days). Wither heights and body condition scores (BCS) were recorded for each cow (18). At the subsequent sampling timepoints only venipuncture and BCS assignment were performed.

### 2.6 Blood analysis

All blood analyses were conducted in the veterinary clinical pathology laboratory at the University of Ljubljana. Hematological parameters were determined using the scil Vet abc Plus™ (Scil animal care company GmbH, Viernheim, Germany). A differential white cell count was performed manually on stained Hemacolor® (Merck KGaA, Dramstadt, Germany) blood smears under an immersion microscope. Biochemical parameters (glutamate dehydrogenase—GLDH [catalog number: GL 441], gamma-glutamyl transferase—GGT [catalog number: GT8320], albumin [catalog number: AB8000], protein [catalog number: TP3869], calcium [catalog number: CA3871], phosphorus [catalog number: PH3872]) were determined using the RX daytona+ analyzer (Randox Laboratories Ltd., Crumlin, United Kingdom). The 25(OH)D concentrations were measured using the VIDAS® 25 OH Vitamin D Total kit [catalog number: 30463] (bioMérieux S.A., Marcy-l’Étoile, France) on the miniVIDAS analyzer (bioMérieux S.A., Marcy-l’Étoile, France).

### 2.7 Statistical analysis

Statistical analysis and plots were generated using R statistical software (v4.2.1; R Core Team 2022, Austria). Normalities of the distributions were assessed using a Shapiro–Wilk test. Depending on the nature of each distribution, either the ANOVA or Kruskal-Wallis tests were used to analyze differences between the groups. Pairwise comparisons of the groups were performed using Tukey’s Honest Significant Difference or the Wilcoxon rank sum test with a Bonferroni *p*-value correction. To assess differences between sampling timepoints, a Repeated Measures ANOVA or Friedman test was used; the Repeated Measures ANOVA accounted for Group, Sampling timepoint and the Group:Sampling timepoint interaction. Cows were included as a random effect. Correlations were analyzed using Spearman’s correlation tests with a Holm p-value correction. The effects of hair/skin black area on 25(OH)D production were assessed using linear regression. The threshold for statistical significance was set at *p* < 0.05.

## 3 Results

All three groups were comparable in terms of parameters measured at the beginning of the study (Table 4). No adverse effects of UV irradiation were observed in any cow during the study.

**Table 4:** Comparison between the Holstein Friesian cow groups at the first sampling timepoint (day 0) with a one-way ANOVA (x̄ ± SEM).

	Shaved (*N* = 16)	Unshaved (*N* = 14)	Control (*N* = 17)	*p*-value
Days in milk	148 ± 24	160 ± 31	153 ± 24	0.98
Milk yield (kg)	30.3 ± 1,5	29.9 ± 2.1	28.2 ± 2.1	0.84
Withers height (cm)	142.5 ± 0,9	141.4 ± 1.2	142.1 ± 1	0.74
BCS	2.9 ± 0,1	2.9 ± 0.1	2.9 ± 0.1	0.92
% black area	59 ± 8.7	51.7 ± 9.5	55.5 ± 8.5	0.71
25(OH)D (ng/mL)	10.8 ± 0.5	11.6 ± 0.7	13.2 ± 0.8	0.07

The shaved group was irradiated daily with 80 J/m^2^. The unshaved group was irradiated with a maximal dose that daily automatic milking time allowed but did not exceed 360 J/m^2^ (range 129–305 J/m^2^). The control group received no irradiation.

### 3.1 25(OH)D production

**Figure 2:**
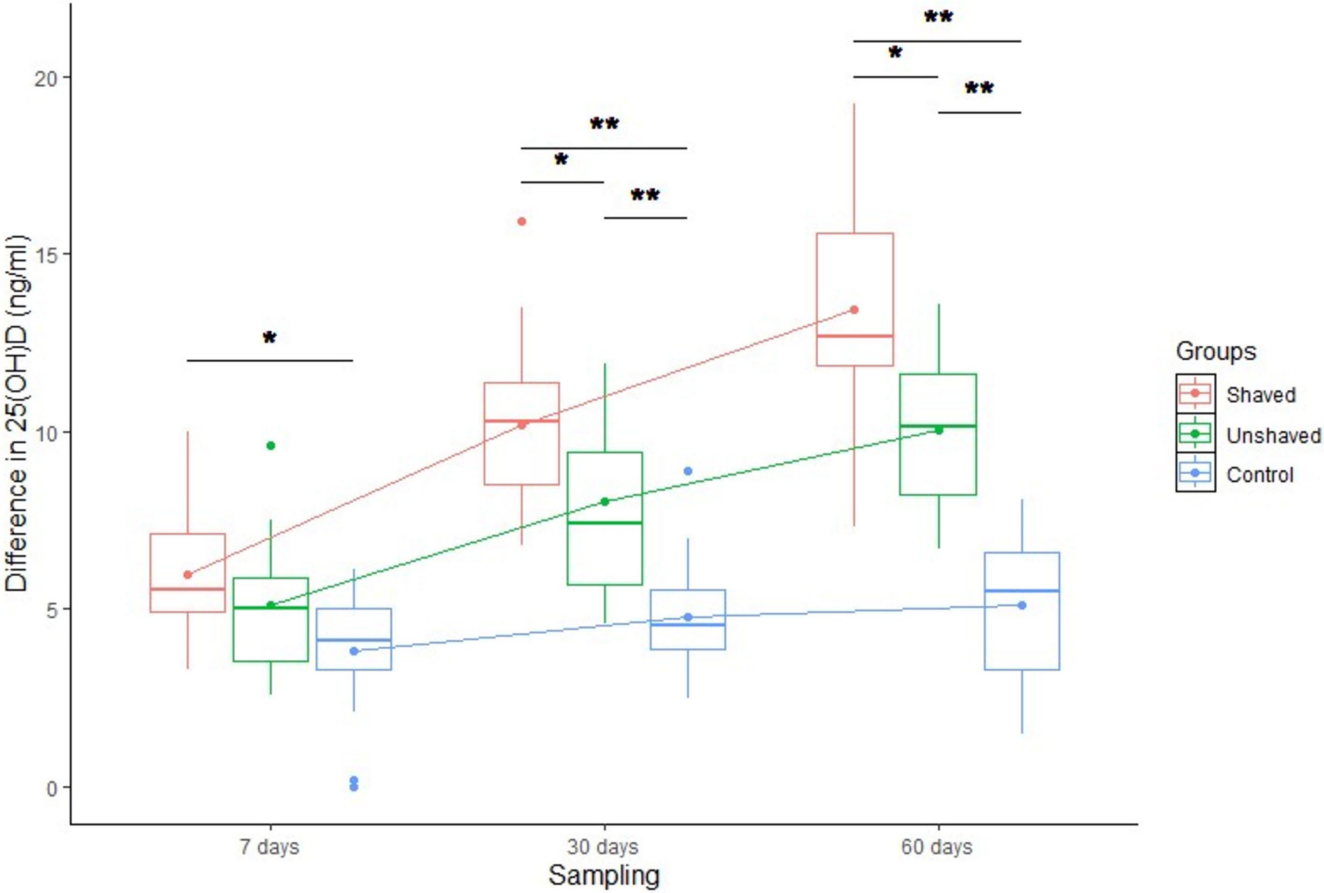
Differences between serum 25-hxdroxyvitamin D (25(OH)D) concentration at day 0 and days 7, 30 and 60 in the three treatment Holstein Friesian cow groups. The shaved group was irradiated daily dose of 80 J/m^2^. The Unshaved group was irradiated with a maximal dose that daily automatic milking time allowed but did not exceed 360 J/m^2^. The control group received no irradiation. *statistically significant difference at *p* < 0.05, **statistically significant difference at *p* < 0.001.

There were significant differences in blood serum 25(OH)D dynamics between groups (*p* < 0.05), sampling timepoints (*p* < 0.001) and group—sampling timepoint interaction (*p* < 0.001; Figure 2; Table 5). Serum 25(OH)D concentrations in the shaven group increased the most (mean 13.4 ng/mL), followed by those from the unshaved group (mean 10 ng/mL). There was a small nonspecific increase in 25(OH)D concentrations of the control group between the first and second sampling timepoints (mean 5.1 ng/mL) after which the concentrations remained fairly constant. The differences between the 25(OH)D concentrations of the groups were significant (Table 5). 73.3% of cows in the irradiated groups exceeded the insufficiency cut-off (20 ng/mL 25(OH)D), and one cow in the shaved group reached optimal 25(OH)D levels (>30 ng/mL 25(OH)D) (1).

**Table 5:** Descriptive statistics of total serum 25-hydroxyvitamin D (25(OH)D) concentrations by cow group and sampling timepoint (¯mean ± SEM).

25(OH)D (ng/mL)	Shaved	Unshaved	Control
0 days	10.8 ± 0.5^a,1^	11.6 ± 0.7^a,1^	13.2 ± 0.8^a,1^
7 days	16.6 ± 0.6^b,1^	16.4 ± 1^b,1^	17 ± 0.8^b,1^
30 days	20.7 ± 0.7^c,1^	19.4 ± 0.9^b,1^	17.8 ± 0.8^b,2^
60 days	24.2 ± 0.9^d,1^	21.5 ± 0.8^c,2^	18.3 ± 0.7^b,3^

Different letters indicate the significant differences between sampling timepoints (differences between rows; *p* < 0.001). Different numbers indicate the significant differences between groups (columns: shaved, unshaved and control; *p* < 0.05). The shaved group was irradiated with a daily dose of 80 J/m^2^. The unshaved group was irradiated with a maximal dose that daily automatic milking time allowed but did not exceed 360 J/m^2^ (range 129–305 J/m^2^). The control group received no irradiation. Group – sampling timepoint interaction was highly significant (*p* < 0.001).

### 3.2 The influence of hair and black area of hair and skin

**Figure 3:**
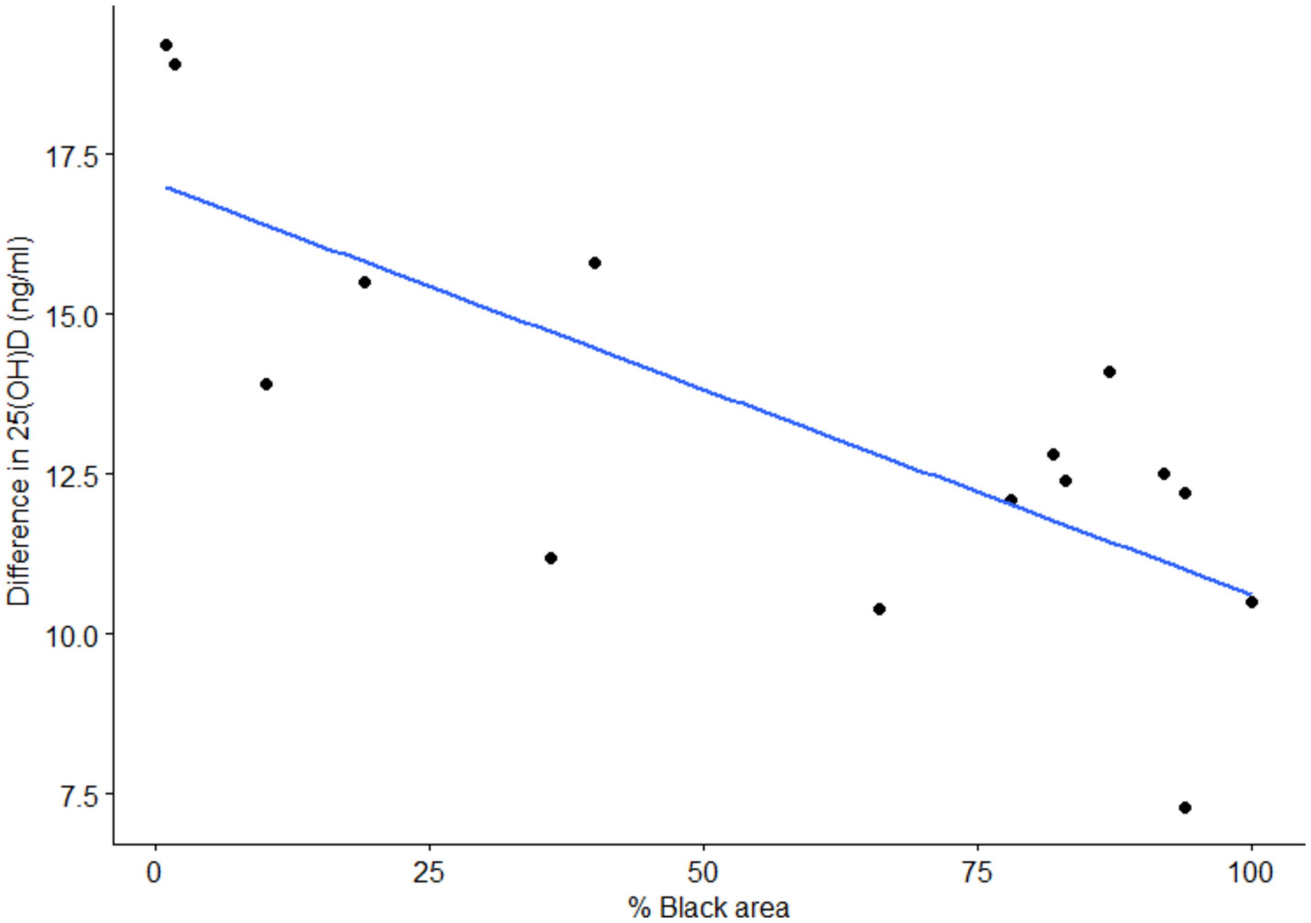
Correlation of the difference in 25-hydroxyvitamin D (25(OH)D) concentration between 1st and 4th sampling with the percent of black skin at the irradiation area in shaved cows. The shaved group was irradiated daily with 80 J/m^2^.

In the shaved group, increases in serum 25(OH)D were negatively correlated with the amount of black surface on the irradiated area (Spearman’s rho = −0.64; *p* = 0.03; Figure 3). This correlation was not observed in the unshaved group (Spearman’s rho = 0.084; *p* = 0.78). Regardless of the percent of black area, the presence of hair also affected vitamin D_3_ production with serum 25(OH)D concentrations of the shaved group increasing by 2.6 ng/mL more than the unshaved group, despite receiving less than half the UV-B radiation dose (*p* = 0.002). The rate of 25(OH)D production was 0.22 ± 0.01 ng/mL per day and 0.17 ± 0.01 ng/mL per day for the shaved and unshaved group, respectively.

### 3.3 Other parameters

We did not observe any significant differences between groups in any of the hematological or biochemical parameters. All the parameters were within the reference range for healthy adult cows (19). Body condition score did not differ between sampling timepoints or groups. Hair length of the unshaved group decreased by 8.4 ± 1.2 mm between the beginning and end of the study (*p* = 0.000014).

### 3.4 Milk yield

**Figure 4:**
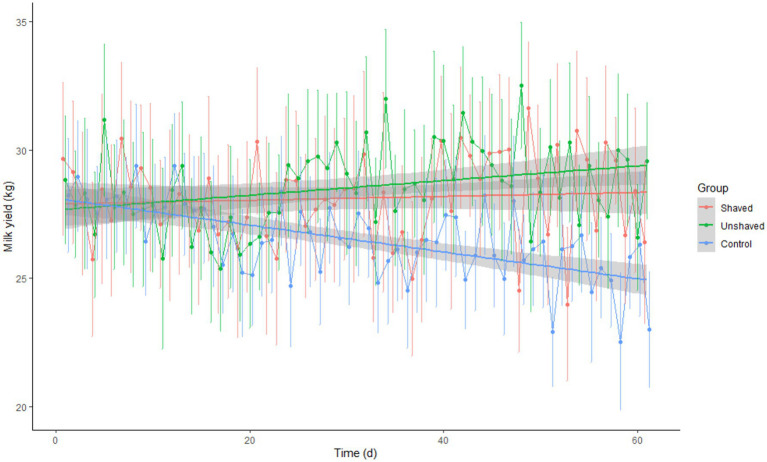
Average daily milk yield ± SEM by cow group in relation to time (days of the experiment). The shaved group was irradiated daily with 80 J/m^2^. The unshaved group was irradiated with a maximal dose that daily automatic milking time allowed but did not exceed 360 J/m^2^ (range 129–305 J/m^2^). The control group received no irradiation.

The difference in milk yield between groups was statistically significant (*p* = 2.96 × 10^−10^; Table 6). Post-hoc analysis showed that both irradiated groups produced more milk than the control group (*p* = 1.2 × 10^−6^ and *p* = 1.6 × 10^−9^). The difference between the irradiated groups was not significant (*p* = 0.79). Milk yield was dependent on time (days of the experiment) and group (*p* < 0.001). However, the correlation between time and milk yield was significant (*p* = 0.0024) only in the control group (Figure 4).

**Table 6:** Milk yield by cow group.

	Shaved	Unshaved	Control
Average daily milk yield	28.7 ± 2.7*	29.4 ± 2.1*	26.5 ± 1.8

The shaved group was irradiated daily with 80 J/m^2^. The unshaved group was irradiated with a maximal dose that daily automatic milking time allowed but did not exceed 360 J/m^2^ (range 129–305 J/m^2^). The control group received no irradiation. *Represent statistically significant difference (*p* < 0.001) compared to the control group.

## 4 Discussion

Exposure to UV-B radiation during milking resulted in increased serum 25(OH)D concentrations. These increases were greatest in the shaved group followed by the unshaved group. An increase in serum 25(OH)D concentration was also observed in the control group between the first and second sampling timepoints. This change was much smaller than that observed in the other two groups and could be due to changes in vitamin D concentration in the feed. However, as feed samples were not collected at the second sampling timepoint, this cannot be demonstrated conclusively. Because of the high 25(OH)D concentrations in the control group at the first timepoint, the increase in 25(OH)D (rather than the absolute 25(OH)D concentration) was used to compare the groups.

At the beginning of the study, some cows had serum 25(OH)D concentrations below 10 ng/mL, classifying them as severely deficient. During the study, most irradiated cows (22/30) exceeded 20 ng/mL which is the cutoff for deficiency, and one cow reached sufficient levels (>30 ng/mL) (1). Some authors suggest that the optimal serum 25(OH)D concentration for cattle should be >40 ng/mL (20). No cow reached this concentration during the current study; however, serum 25(OH)D concentration did not plateau implying that if the study had been extended, more cows could have reached sufficient or even optimal levels. In addition, the use of a higher irradiation dose for the unshaved group may further improve vitamin D_3_ production in these animals as this process is linearly dose dependent (7, 8).

The rate of 25(OH)D production was 0.22 ± 0.01 ng/mL per day and 0.17 ± 0.01 ng/mL per day for the shaved and unshaved group, respectively. A UV wavelength of 295 nm was used in this study as it is believed to be the optimal UV wavelength for pre-vitamin D_3_ production. Furthermore, narrowband UV-B from LED lights has been shown to be more effective in pre-vitamin D_3_ production than the broadband UV light from the sun (21). In the shaved group, the rate of 25(OH)D production was below the maximal 25(OH)D increase per day in cattle (1 ng 25(OH)D/mL/day) reported by Hymøller et al. (13). It should be noted that in this current study, only the backs of the cows were irradiated. In contrast, Hymøller et al. exposed cows to a higher UV irradiation dose (1,200 J/m^2^), and the backs and hindquarters of the cows were irradiated; irradiating a larger skin surface and using a higher dose could therefore increase 25(OH)D concentration more rapidly. The study by Hymøller et al. also reported that vitamin D_3_ production could not be further increased by using doses above 1,200 J/m^2^. This is most likely because continued exposure results in the synthesis of other photoproducts when using broad-spectrum UV including solar UV (8). However, it is hypothesized that the rate of 25(OH)D production would continue to increase if a larger skin surface area was irradiated and more optimal UV wavelengths used (21).

Cows produce vitamin D_3_ over their entire skin surface not just in the areas scarcely covered by hair (3). However, cattle hair is known to reduce UV transmission to the skin (16). In this study, shaved cows had significantly higher increases in their 25(OH)D concentrations compared to their unshaved counterparts, although the former received a lower dose. Studies in sheep have also shown that shorn sheep have higher 25(OH)D concentrations than their unshorn flock mates (22, 23).

In the shaved group, the amount of 25(OH)D produced was negatively correlated with the percentage of black skin in the irradiated area (*p* = 0.009). To the best of the authors’ knowledge, this is the first report of a correlation between black skin area and cutaneous vitamin D_3_ production in cattle. Interestingly we found no correlation between black hair area and cutaneous 25(OH)D production in the unshaved group. This could be because the hair length changed during the study or because of a difference in hair characteristics other than pigment between white and black hair (24). Other studies have also failed to find a correlation between the ratio of black to white hair and 25(OH)D production in cows (7, 12, 13). Hair pigment was found to influence 25(OH)D levels in alpacas (25) and calves (14). However, the hair characteristics of alpacas and cattle differ and the calves in the latter study belonged to a different species (*Bos indicus*) to the cows in this study (*Bos taurus*) and therefore might also have different hair characteristics.

Milk production may be correlated with vitamin D supplementation. Some studies have found an association between blood 25(OH)D levels and milk yield (26, 27). However, Golder et al. (28) did not find any effect of 25(OH)D supplementation during lactation on milk yield. In this current study, milk yield was found to be higher in the irradiated groups than the control group however no statistically significant difference was found between the two irradiated groups even though the shaved group had higher 25(OH)D concentrations. Therefore, the observed differences in milk yield could be attributed to a poorer performance in some of the control cows with insufficient levels of 25(OH)D or may have been influenced by culling of the animals in the irradiated groups (1 in the shaved and 3 in the unshaved). This suggests that there must have been other more significant influences on milk yield or that serum 25(OH)D influence milk yield up to a certain concentration after which its effect plateaus.

This study was conducted using one breed of cattle from a single herd over a 60-day period therefore more studies are needed to see whether these results can be extrapolated to the wider dairy cattle population. Future studies should be conducted on multiple herds and different breeds of cattle in order to assess how different hair lengths, thickness and pigmentation might affect cutaneous vitamin D production. A longer study duration would allow the long-term effects of UV-B exposure on cow health, such as squamous cell carcinoma incidence, to be assessed. The optimal wavelengths of UV light needed for pre-vitamin D_3_ production in cattle are still unknown and might differ between breeds. Higher doses or a greater surface of irradiation could be tested to achieve a higher 25(OH)D increase.

In conclusion, this study has demonstrated that artificial UV-B irradiation can be used to stimulate autogenous vitamin D_3_ production during automatic milking. Cutaneous production is influenced by the percentage of black skin on the irradiation site but not the percentage of black hair area. The presence of hair does impair cutaneous vitamin D_3_ production.

## Data Availability

The original data presented in the study are publicly available and can be found here: https://repozitorij.uni-lj.si/IzpisGradiva.php?id=166415.

## References

[1] Nelson CD Lippolis JD Reinhardt TA Sacco RE Powell JL Drewnoski ME. Vitamin D status of dairy cattle: outcomes of current practices in the dairy industry. J Dairy Sci. (2016) 99:10150–60. doi: 10.3168/jds.2016-11727 27743666

[2] Hodnik JJ Ježek J Starič J. A review of vitamin D and its importance to the health of dairy cattle. J Dairy Res. (2020) 87:84–7. doi: 10.1017/S0022029920000424, PMID: 33213577

[3] Hymøller L Jensen SK. Vitamin D3 synthesis in the entire skin surface of dairy cows despite hair coverage. J Dairy Sci. (2010) 93:2025–9. doi: 10.3168/jds.2009-2991, PMID: 20412916

[4] Dusso AS Brown AJ Slatopolsky E. Vitamin D. American J Physiol Renal Physiol American Physiolog Society. (2005) 289:F8–F28. doi: 10.1152/ajprenal.00336.2004 15951480

[5] Hymøller L Jensen SK. Vitamin D2 impairs utilization of vitamin D3 in high-yielding dairy cows in a cross-over supplementation regimen. J Dairy Sci. (2011) 94:3462–6. doi: 10.3168/jds.2010-4111, PMID: 21700032

[6] Duffy SK O’Doherty JV Rajauria G Clarke LC Hayes A Dowling KG. Vitamin D-biofortified beef: a comparison of cholecalciferol with synthetic versus UVB-mushroom-derived ergosterol as feed source. Food Chem. (2018) 256:18–24. 29606435 10.1016/j.foodchem.2018.02.099

[7] Hymøller L Jensen SK. 25-hydroxycholecalciferol status in plasma is linearly correlated to daily summer pasture time in cattle at 56°N. Br J Nutr. (2012) 108:666–71. doi: 10.1017/S0007114511005964, PMID: 22309951

[8] MacLaughlin JA Anderson RR Holick MF. Spectral character of sunlight modulates photosynthesis of. Science. (1979) 216:1001–3. doi: 10.1126/science.6281884 6281884

[9] Casas E Lippolis JD Kuehn LA Reinhardt TA. Seasonal variation in vitamin D status of beef cattle reared in the Central United States. Domest Anim Endocrinol. (2015) 52:71–4. doi: 10.1016/j.domaniend.2015.03.003, PMID: 25917139

[10] Jakobsen J Saxholt E. Vitamin D metabolites in bovine milk and butter. J Food Compos Anal. (2009) 22:472–8. doi: 10.1016/j.jfca.2009.01.010

[11] Engelsen O. The relationship between ultraviolet radiation exposure and vitamin D status. Nutrients. (2010) 2:482–95. doi: 10.3390/nu2050482, PMID: 22254036 PMC3257661

[12] Jakobsen J Jensen SK Hymøller L Andersen EW Kaas P Burild A. Short communication: artificial ultraviolet B light exposure increases vitamin D levels in cow plasma and milk. J Dairy Sci. (2015) 98:6492–8. doi: 10.3168/jds.2014-9277, PMID: 26117346

[13] Hymøller L Jensen SK Kaas P Jakobsen J. Physiological limit of the daily endogenous cholecalciferol synthesis from UV light in cattle. J Anim Physiol Anim Nutr (Berl). (2017) 101:215–21. doi: 10.1111/jpn.12540, PMID: 27421247

[14] Callaby R Hurst E Handel I Toye P de C BBM Mellanby RJ. Determinants of vitamin D status in Kenyan calves. Sci Rep. (2020) 10:20590. doi: 10.1038/s41598-020-77209-5, PMID: 33239727 PMC7688966

[15] Dittmer K Thompson K. Vitamin D metabolism and rickets in domestic animals: a review. Vet Pathol. (2011) 48:389–407. doi: 10.1177/0300985810375240 20634407

[16] Hodnik JJ Jankovec M Ježek J Krušič Ž Mitterhofer S Starič J. Minimal erythema dose determination in Holstein Friesian cattle. Front Vet Sci. (2021) 8:8. doi: 10.3389/fvets.2021.757452 PMC8591259 34790714

[17] Dowdy JC Sayre RM Holick MF. Holick’s rule and vitamin D from sunlight. J Steroid Biochem Mol Biol. (2010) 121:328–30. doi: 10.1016/j.jsbmb.2010.04.002, PMID: 20398766

[18] Ferguson JD Galligan DT Thomsen N. Principal descriptors of body condition score in Holstein cows. J Dairy Sci. (1994) 77:2695–703. doi: 10.3168/jds.S0022-0302(94)77212-X, PMID: 7814740

[19] Divers TJ Peek SF. Rebhun’s diseases of dairy cattle. Third edition. St Louis: Elsevier. (2018) 14–5.

[20] Nelson CD Merriman KE. “Vitamin D metabolism in dairy cattle and implications for dietary requirements.” In: *Florida ruminant Nutition symposium*. (2014). p. 78–90. Available at: https://animal.ifas.ufl.edu/apps/dairymedia/rns/2014/nelson.pdf

[21] Kalajian TA Aldoukhi A Veronikis AJ Persons K Holick MF. Ultraviolet B light emitting diodes (LEDs) are more efficient and effective in producing vitamin D3 in human skin compared to natural sunlight. Sci Rep. (2017) 7:6–13. doi: 10.1038/s41598-017-11362-2 28904394 PMC5597604

[22] Quarterman J Dalgarno AC Adam A. Some factors affecting the level of vitamin D in the blood of sheep. Br J Nutr. (1964) 18:79–89. doi: 10.1079/BJN19640008, PMID: 14112972

[23] Hidiroglou M Karpinski K. Providing vitamin D to confined sheep by oral supplementation vs ultraviolet irradiation. J Anim Sci. (1989) 67:794–802. doi: 10.2527/jas1989.673794x, PMID: 2542212

[24] Udo HMJ. Hair coat characteristics in Friesian heifers in the Netherlands and Kenya: Experimental data and a review of literature. Weningen: H. Veenman & zonen B.V (1978). 136 p.

[25] Judson G McGregor B Partington D. Factors associated with low vitamin D status of Australian alpacas. Aust Vet J. (2008) 86:486–90. doi: 10.1111/j.1751-0813.2008.00367.x 19076772

[26] Poindexter MB Zimpel R Vieira-Neto A Husnain A Silva ACM Faccenda A. Effect of prepartum source and amount of vitamin D supplementation on lactation performance of dairy cows. J Dairy Sci. (2023) 106:974–89. doi: 10.3168/jds.2022-22388, PMID: 36526464

[27] Martinez N Rodney RM Block E Hernandez LL Nelson CD Lean IJ. Effects of prepartum dietary cation-anion difference and source of vitamin D in dairy cows: lactation performance and energy metabolism. J Dairy Sci. (2018) 101:2544–62. doi: 10.3168/jds.2017-13739, PMID: 29274965

[28] Golder HM McGrath J Lean IJ. Effect of 25-hydroxyvitamin D3 during prepartum transition and lactation on production, reproduction, and health of lactating dairy cows. J Dairy Sci. (2021) 104:5345–74. doi: 10.3168/jds.2020-18901, PMID: 33663856

